# XingNaoJing, prescription of traditional Chinese medicine, prevents autophagy in experimental stroke by repressing p53-DRAM pathway

**DOI:** 10.1186/s12906-015-0882-2

**Published:** 2015-10-19

**Authors:** Gang Wei, YueChun Huang, Fei Li, FeiJian Zeng, YiWei Li, RuDong Deng, YingTao Lai, JianHong Zhou, GuiHua Huang, DongFeng Chen

**Affiliations:** Research & Development of New Drugs, Guangzhou University of Chinese Medicine, Guangzhou, 510006 China; Molecular medicine, Guangzhou University of Chinese Medicine, Guangzhou, 510006 China; The first affiliated hospital of GuangXi university of Chinese medicine, Dongge Road No. 89-9, Nanning, 530023 China; Department of Anatomy, Guangzhou university of Chinese medicine, Jichang Road No.12, Guangzhou, 510405 China

**Keywords:** Xingnaojing, Autophagy, p53, Damage-regulated autophagy modulator, Stroke

## Abstract

**Background:**

Xingnaojing (XNJ), a well known prescription in traditional Chinese medicine, has been used for treatment of stroke in China. However, the effects and mechanisms of XNJ on autophagy are not clear. Here, we used the cell models of autophagy induced by serum-free condition and ischemia stroke in rats to further investigate whether the p53-DRAM pathway is involved in the effects of XNJ on autophagy.

**Methods:**

We used the cell model of autophagy induced by serum-free condition and the rat model of ischemia caused by a middle cerebral artery occlusion (MCAO). The effects of XNJ on p53 transcriptional activity of PC12 cells were evaluated by the luciferase activity assay. The mRNA levels and the expression of p53 and its target autophagy gene DRAM (damage-regulated autophagy modulator) were analyzed respectively by Quantitative-RTPCR and Western blot assay. The activation of autophagy was detected by the levels of autophagy markers, microtubule associated protein light chain 3 (LC3) and p62 by Immunofluorescence and Western blot. p53 inhibitor was used to determine whether p53 is responsible for the effects of XNJ on preventing autophagy.

**Results:**

The assay for luciferase activity of p53 promoter indicated that XNJ inhibited p53 transcriptional activity. XNJ reduced the expression of p53 and its target autophagy gene DRAM (damage-regulated autophagy modulator) in serum-free condition PC12 cells and the cortex in MCAO rats. XNJ reduced autophagy of PC12 cells induced by serum-free condition and the cortex in MCAO rats. Furthermore, suppression of p53 by p53 inhibitor significantly reduced the effects of XNJ on the autophagy of PC12 cells in serum-free condition.

**Conclusion:**

XNJ prevents autophagy in experimental stroke by repressing p53/DRAM pathway. Our findings are therefore of considerable therapeutic significance and provide the novel and potential application of XNJ for the treatment of brain diseases.

## Background

Autophagy, which plays key roles in the digestion of most cytosolic and aggregated or misfolded proteins in brain [1], plays an important part in both cell survival and cell death [2]. Many studies have shown that autophagy is the predominant mode of neuronal death in stroke [3–5]. Therefore, it is essential to investigate the mechanisms underlying the prevention of autophagy associated with such destructive diseases. p53 is a tumor suppressor protein that activates transcriptional programs under various types of cellular stress [6]. A link between autophagy and the regulation of p53 processing has been suggested, the regulation of p53 represents a crucial step in the molecular cascade of events leading to autophagy. p53 in the regulation of autophagy is controlled by its subcellular localization [7]. Nuclear p53 stimulates autophagy in a transcription-dependent fashion [8, 9], while cytoplasmic p53 protein represses autophagy in a transcription-independent way [10]. Recently, DRAM (damage-regulated autophagy modulator) is a lysosomal protein that is not only a new p53 target which modulates autophagy, but also for p53’s ability to induce programmed cell death [8]. Consequently, it has been proposed that p53 is an important pharmacological target of intersection for autophagy.

XingNaoJing (XNJ), is one of a hundred traditional Chinese medicinal (TCM) agents used clinically in China for the treatment of stroke, and has approval from the Chinese National Drug Administration [11]. XNJ consists of four Chinese herbs: *Moschus*, a dry substance secreted by a gland in the sub-umbilical sac of the male musk deer; Radix *Curcumae*, the dried roots of *Curcuma aromatica* Salisb and *C. zedoaria* (Berg) Rosc., family Zingiberaceae; Fructus *Gardeniae*, the fruit of *Gardenia jasminoides* Ellis var. *radicans* (Thunb.) Makino, family Rubiaceae; and crystals from the evaporated exudate of the trunk of *Dryobalanops aromatica* Gaertn. f., family Dipterocarpceae. Clinical trials have reported that XNJ can reduce brain injury and enhance functional recovery after stroke [12]. Pharmacological studies have demonstrated that XNJ has neuroprotective effects in cell and animal models of stroke [13, 14]. Recent studies have shown the neuroprotective effect of a herb pair of XNJ on ischemia stroke in rats [15]. However, the effects and mechanisms of XNJ on the autophagy are not clear. Here, we used the cell models of autophagy induced by serum-free condition and ischemia stroke in rats to further investigate whether the p53-DRAM pathway is involved in the effects of XNJ on autophagy.

## Methods

### Animals and materials

Sprague–Dawley (SD) rats were obtained from the animal centre of Guangzhou University of Chinese Medicine. All animals were given humane care according to the guidelines set by the Care of Experimental Animals Committee of Guangzhou University of Chinese Medicine, and the study was submitted to, and approved by, our institutional ethics committee. Dulbecco’s modified Eagle’s medium (DMEM), fetal bovine serum (FBS), Lipofectamine 2000 regent and nerve growth factor were purchased from Invitrogen (California, USA); microtubule associated protein light chain 3 (LC3), p62, p53 and DRAM antibody were provided by Santa Cruz Biotechnologies (Santa Cruz, CA, USA); chemicals such as dimethyl sulphoxide (DMSO) and other reagents were also obtained from Sigma; XingNaoJing injection (batch number: 140704, 141219) was bought from Shanhe Pharmaceutical Co., Ltd (Wuxi, China). The p53 promoter-Luc vector, the pGL3-Basic Vector and pRL-TK plasmid were kindly provided to our laboratory by Dr. Huang Qilai and Dr. Chen Yuan (State Key Laboratory of Pharmaceutical Biotechnology, Nanjing University). Dual Luciferase Assay Kit (Promega, Wisconsin, USA).

### Culture of PC12 cells

Partially differentiated PC12 cells provided by Shanghai Cellular Institute of China Scientific Academy (Shanghai, China), originated from rat pheochromocytoma, were grown to confluence in containing DMEM (1000 mg/l glucose) supplemented with 5 % FBS, 10 % horse serum, and a mixture of 1 % of penicillin/streptomycin/nystatin. Cell cultures were incubated at 37 °C in a humid 5 % CO_2_/ 95 % air environment. PC12 cells were differentiated with 100 ng/ml nerve growth factor for 7 days.

### Cell transfection and assay for luciferase activity

For luciferase activity assays, PC12 cells were transfected with p53 promoter reporter construct by Lipofectamine 2000 regent, plasmid for pRL-TK was cotransfected to normalize the variations in transfection efficiency, and then stimulated with serum or serum-free condition for 6, 12, 24 and 36 h respectively. PC12 cells were transfected with p53 promoter reporter construct and then stimulated with serum or serum-free condition for 12 h in the absence or presence of pifithrin α at 0.5, 5 and 50 μM or XNJ at 50 μl/ml and 150 μl/ml. 10 μl of cell lysate was assayed first for firefly luciferase and then for Renilla luciferase activity. The absolute values of firefly luminescence were normalized to those of Renilla, and the ratios were presented as the relative luciferase units (RLU).

The cell models of autophagy induced by serum-free condition were used. PC12 cells were cultured in serum-free condition and stimulated with p53 inhibitor in the absence or presence of XNJ for 48 h, the levels of p53 and its target autophagy gene DRAM (damage-regulated autophagy modulator) mRNA were analyzed by Quantitative-RTPCR. Western blot assay was performed to detect the autophagy activity, levels of LC3 and p62 proteins. Since both the ratio of LC3-II to LC3-I and the amount of LC3-II could be used to monitor autophagosome formation, LC3-II (approximately 16 kDa) was used for densitometry quantitation in our study.

### Middle cerebral artery occlusion model and treatment schedules

As described previously, the middle cerebral artery occlusion (MCAO) was induced in adult male Sprague–Dawley rats (280–300 g) using the intraluminal filament technique [16]. Rats were anesthetized with an intraperitoneal injection of 10 % chloral hydrate at a dose of 0.33 mL/100 g. A midline neck incision was made; the right common carotid artery and external carotid artery were isolated. A nylon filament was inserted into the middle cerebral artery and maintained for 120 min. Reperfusion was achieved by withdrawing the suture after 120 min of occlusion. After operation, rats were transferred to a temperature-controlled chamber to maintain body temperature at 37.5 °C. 1 h after reperfusion (0 d), rats were scored for neurological function according to a scoring system reported by Longa [16]. After neurological evaluation of MCAO rats, treatment schedules were performed.

MCAO rats were randomly divided into 3 groups (*n* = 30/per group): MCAO + vehicle group, MCAO + XNJ 1 ml/day and MCAO + XNJ 3 ml/day. XNJ was administered by vein injection after 2 h of reperfusion and again administrated with the same dosage daily for 1 day. The MCAO group received the same volume of vehicle. After the treatment of XNJ for 1 day, brains were removed and placed on ice. Seven brains were removed and fixed in 10 % buffered formalin phosphate for 24 to 48 h for paraffin embedding. Serial coronal frontal cortex sections (5 μm) were cut and every tenth section was systematically assigned to a series of sections, yielding a total of 10 series. One series of sections was saved for LC3 immunohistochemical analysis. For PCR and Western blot analysis, six ipsilateral cortices were rapidly removed and placed on ice. The cortices were stored at −80 °C for analysis.

### Immunofluorescence

Immunofluorescence for LC3 was conducted following a two-step protocol. Briefly, slides from cultured PC12 cells and coronal frontal cortex sections of MCAO rats were successively incubated with LC3 antibody and the second antibody (FITC-conjugated IgG). Subsequently, the sections were incubated with propidium iodide. The control, with the identical procedure, was stained with non-immune serum instead of the primary antibody. The percentage of positive cells was assessed as the ratio of positive cells to total cells in the fields.

### Quantitative real-time reverse transcription-polymerase chain reaction analysis

RNA was isolated as a standard protocol for quantitative real-time reverse transcription polymerase chain reaction analysis. Cells were synchronized overnight in serum-free condition and then were stimulated with a different dose of XNJ, ranging 50–150 μl/ml for 48 h. After stimulation, cells were washed with PBS and total cellular RNA was extracted using Trizol reagent (Invitrogen, Carlsbad, CA, USA) according to the manufacturer’s recommendation. 5 μl of the total RNA was reverse-transcribed into cDNA (RT-PCR reagent, QIAGEN, Hilden, Germany), and was amplified by fluorescent quantity PCR using the ABI PRISM 7900HT Sequence Detection System (Applied Biosystems, Foster City, CA, USA). The fluorescent quantity PCR condition was a pre-denaturation at 93 °C for 2 min, then 40–45 cycles of 93 °C for 45 s and 55 °C for 1 min.

### Western blot analysis

Proteins extracted from cultured PC12 cells, as well as the cortices of MCAO rats were separated by 10 % SDS–PAGE, electrophoretically transferred to nitrocellulose (Bio-Rad, California, USA). The blot was probed with LC3, p62, p53 and DRAM antibody respectively, followed by a second antibody labeled with horseradish-peroxidase at room temperature for 90 min. Bands were visualized with an enhanced chemiluminescence kit according to the manufacturer’s protocol. Densitometry quantitation was used to analyze with ImageJ Software (National Institutes of Health, Bethesda, MD, USA).

### Data analysis

All data were expressed as mean ± standard error of the mean (SEM) for each group. Analysis of variance was carried out using SPSS 10.0 for Windows software. Effects were considered to be significant at P values less than 0.05.

## Results

### XNJ inhibits p53 transcriptional activity

**Fig. 1:**
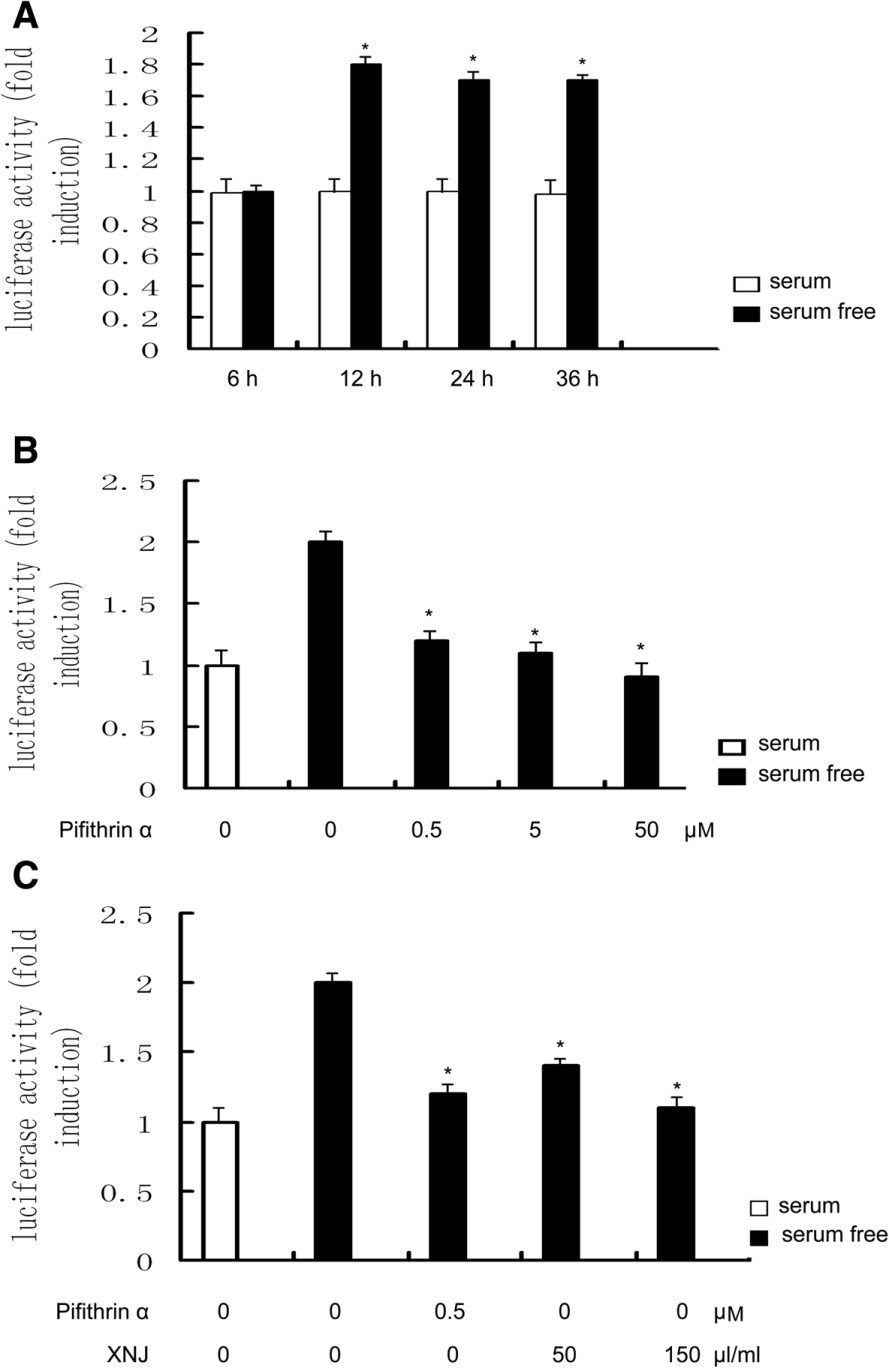
XNJ inhibits p53 transcriptional activity. **a** Serum-free condition induced the p53 transcriptional activity. PC12 cells were transfected with p53 promoter reporter construct and then stimulated with serum or serum-free condition for 6, 12, 24 and 36 h respectively. **b** p53 inhibitor, pifithrin α, inhibited p53 transcriptional activity. PC12 cells were transfected with p53 promoter reporter construct and then stimulated with serum or serum-free condition for 12 h in the absence or presence of pifithrin α. **c** XNJ inhibited p53 transcriptional activity. PC12 cells were transfected with p53 promoter reporter construct and then stimulated with serum or serum-free condition for 12 h in the absence or presence of XNJ. Luciferase activity was determined in cell lysates and normalized to *Renilla* activity. Results are representative of three independent experiments in duplicate

Serum-free condition induced the p53 transcriptional activity. PC12 cells were transfected with p53 promoter reporter construct and then stimulated with serum or serum-free condition for 6, 12, 24 and 36 h respectively. As shown in Fig. 1a, increase in p53 promoter activity of PC12 cells in the serum-free condition was observed at 12 h, 24 h and 36 h, but not 6 h, suggesting that serum-free condition increases the p53 promoter activity in a time dependent manner. We evaluated the dose effects of p53 inhibitor on the p53 promoter activity of PC12 cells. PC12 cells were transfected with p53 promoter reporter construct and then stimulated with serum or serum-free condition for 12 h in the absence or presence of p53 inhibitor, pifithrin α. As shown in Fig. 1b, pifithrin α inhibits p53 transcriptional activity in a dose dependent manner. We also evaluated the dose effects of XNJ on the p53 promoter activity of PC12 cells. As seen in Fig. 1c, increase in p53 promoter activity of PC12 cells in the serum-free condition was reduced by XNJ in a dose-dependent manner, suggesting that XNJ inhibits p53 transcriptional activity.

### XNJ reduced the expression of p53 in serum-free condition PC12 cells

**Fig. 2:**
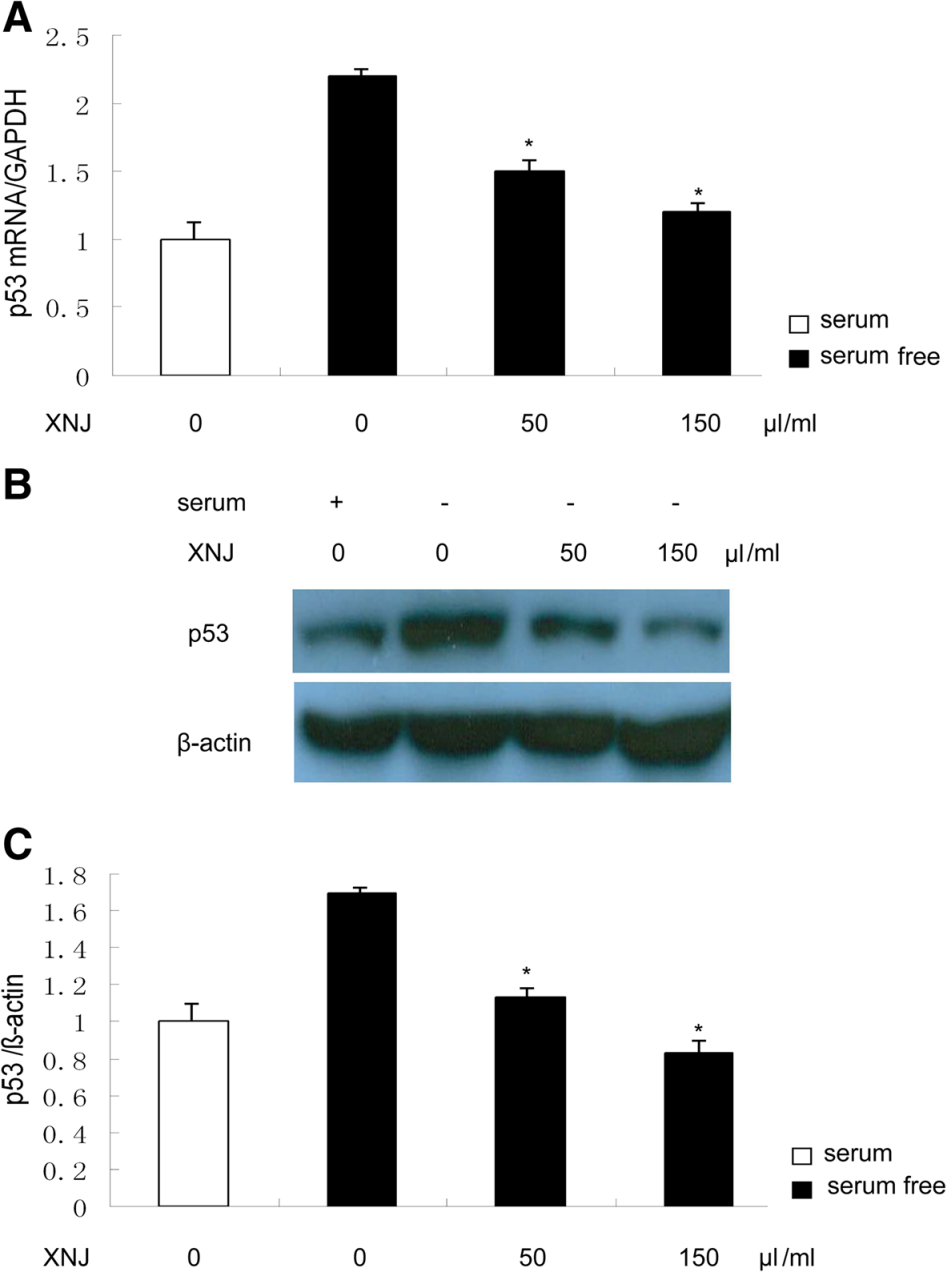
XNJ inhibits p53 expression in serum-free condition PC12 cells. PC12 cells were treated with serum or serum-free condition for 48 h in the absence or presence of XNJ. **a** p53 mRNA was down-regulated by XNJ. The levels of p53 mRNA were analyzed by Q-PCR. **b** p53 expression was down-regulated by XNJ. After XNJ treatment, the cells were lysed for Western blot analysis using an enhanced chemiluminescence system. **c** The density of p53/β-actin band among the experimental groups was compared. Data are mean ± SEM of values obtained from three independent experiments. *, *p* < 0.05 compared with serum-free condition

To further test the effects of XNJ on the p53 expression of PC12 cells in serum-free condition, PC12 cells were treated with serum or serum-free condition for 48 h in the absence or presence of XNJ. As shown in Fig. 2a, increase in p53 mRNA of PC12 cells in the serum-free condition was observed at 48 h, suggesting that serum-free condition increases the p53 mRNA. In contrast, the p53 mRNA expression was attenuated in the serum-free condition treated with XNJ in a dose-dependent manner. As Western blot analysis shown in the Fig. 2b,c, when PC12 cells were treated in serum-free condition alone, the expression of P53 increased remarkably; while XNJ at low concentration (50 μl/ml) decreased p53 levels, and this decrease was further enhanced by high concentration (150 μl/ml), suggesting that XNJ elicits a dose-dependent reduce in p53 levels.

### XNJ inhibits p53 target autophagy gene in serum-free condition PC12 cells

**Fig. 3:**
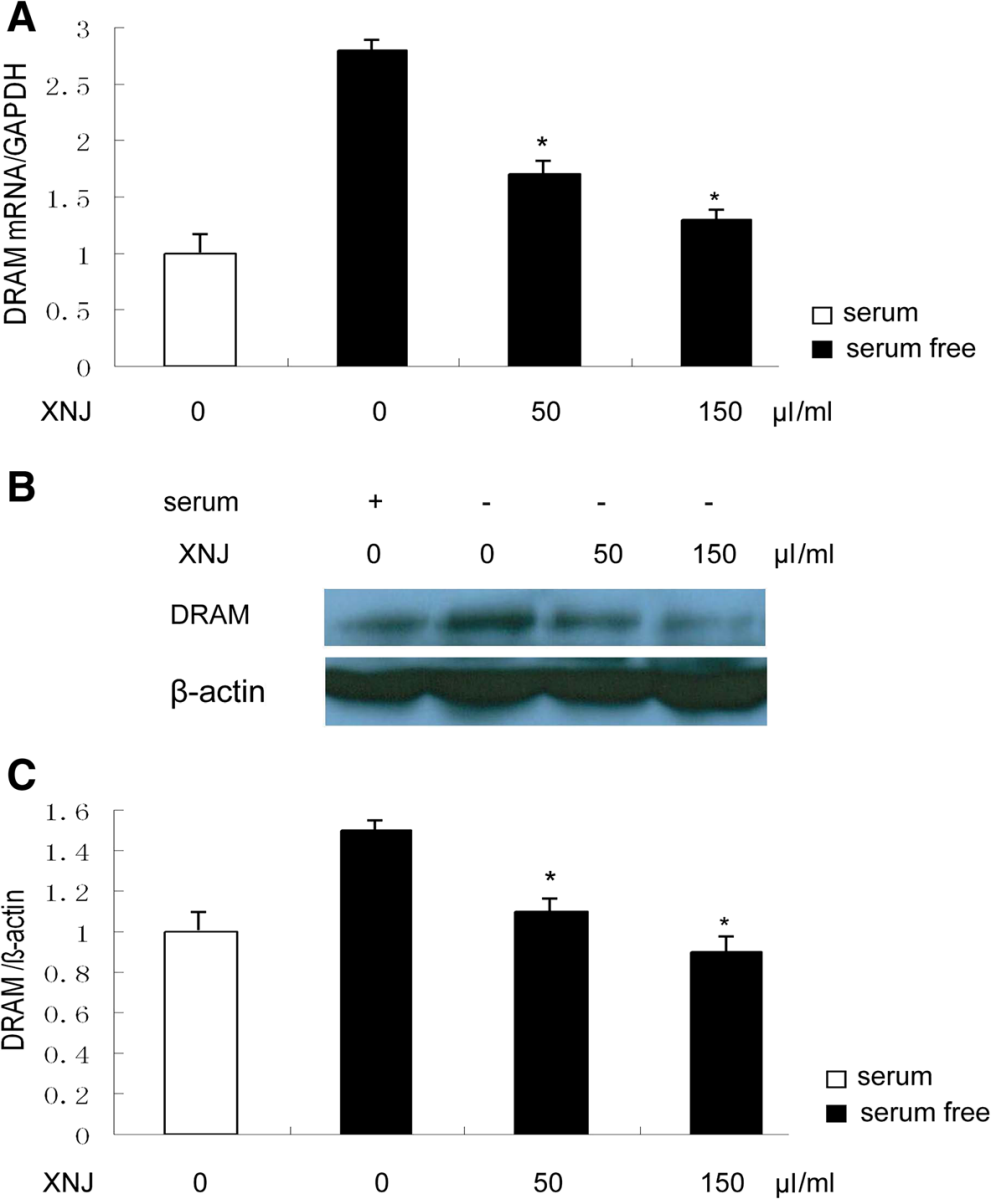
XNJ inhibits p53 target autophagy gene expression in serum-free condition PC12 cells. PC12 cells were treated with serum or serum-free condition for 48 h in the absence or presence of XNJ. **a** DRAM mRNA was down-regulated by XNJ. The levels of DRAM mRNA were analyzed by QRT-PCR. **b** DRAM expression was down-regulated by XNJ. After XNJ treatment, the cells were lysed for Western blot analysis using an enhanced chemiluminescence system. **c** The density of DRAM/β-actin band among the experimental groups was compared. Data are mean ± SEM of values obtained from three independent experiments. *, *p* < 0.05 compared with serum-free condition

To demonstrate the involvement of p53 in autophagy activity of XNJ, measurement of autophagy gene DRAM mRNA expression, target gene of p53 was performed. RT-PCR analysis demonstrated that treatment of PC12 cells with XNJ significantly decreased the DRAM mRNA expression, compared with the level observed in serum-free condition (Fig. 3a). In order to further characterize the effects of XNJ, we examined the changes in DRAM level. Western blot analysis was performed at 48 h after the PC12 cells in serum-free condition were treated to measure the DRAM expression (Fig. 3b). The group of the PC12 cells in serum-free condition treated with vehicle showed a robust increase in the expression of DRAM. In contrast, the DRAM expression was attenuated by XNJ. As shown in the Fig. 3c, XNJ treatment significantly reduced the DRAM expression level in the PC12 cells in serum-free condition (from 1.53 ± 0.4 % to 1.10 ± 0.3 % and 0.90 ± 0.2 %), compared with the group treated with vehicle (*P* < 0.05). Taken together, these results suggest that XNJ reduced DRAM expression of the PC12 cells in serum-free condition.

### XNJ reduced autophagy of the PC12 cells in serum-free condition

**Fig. 4:**
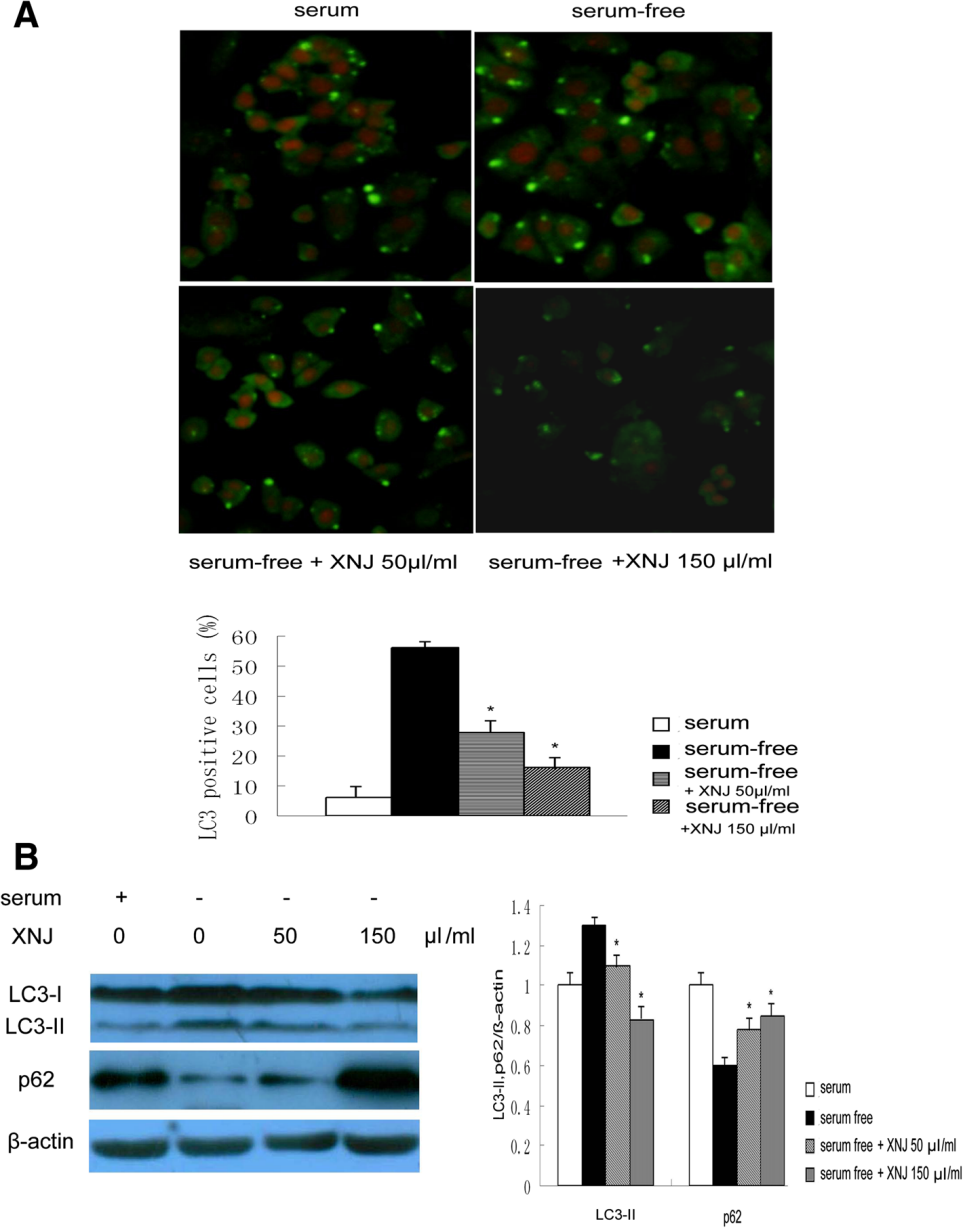
XNJ reduced autophagy of PC12 cells induced by serum-free condition. PC12 cells were treated with serum or serum-free condition for 48 h in the absence or presence of XNJ. **a** The LC3-positive cells were analyzed using immunofluorescence (upper panel, original magnification, 200×), and comparison of the percentage of LC3-positive cells among the experimental groups was showed (lower panel). **b** The expression levels of LC3 and p62 were analyzed using Western blot assay (left panel). The density of LC3-II and p62/β-actin band was compared among the experimental groups (right panel). Data are mean ± SEM of values obtained from three independent experiments. *, *p* < 0.05 compared with serum-free condition

To explore the effects of XNJ on autophagy, LC3 was used to identify autophagy. There was an increase in LC3-positive cells of PC12 cells in serum-free condition, whereas PC12 cells in serum-free condition treated with XNJ showed very few LC3-positive cells. Quantitative analysis of LC3-positive cells (Fig. 4a) revealed that LC3-positive cells in the serum-free condition reduced from 56 ± 2.4 % in the group treated with vehicle to 28 ± 1.9 %, 16 ± 2.2 % in the group treated with XNJ. We examined the changes in LC3 levels. Western blot analysis was performed at 48 h after the PC12 cells in serum-free condition were treated to measure the LC3 expression (Fig. 4b). The group of the PC12 cells in serum-free condition treated with vehicle showed a robust increase in the expression of LC3-II. In contrast, the LC3-II expression was attenuated in the PC12 cells in serum-free condition treated with XNJ. As shown in the Fig. 4b, the XNJ treatment significantly reduced the LC3-II expression level of the PC12 cells in serum-free condition (from 1.31 ± 0.8 to 1.0 ± 0.6 and 0.8 ± 0.4), compared with the group treated with vehicle (*P* < 0.05). To further examine the effects of XNJ on autophagy flux, western blot analysis was performed to measure the p62 levels. The group of the PC12 cells in serum-free condition treated with vehicle showed a robust decrease in p62 levels. In contrast, a significant accumulation of p62 protein was seen in the PC12 cells in serum-free condition treated with XNJ (Fig. 4b), suggesting that autophagy flux was reduced by XNJ. Taken together, these results suggest that XNJ reduced autophagy of the PC12 cells in serum-free condition.

### p53 is responsible for the effects of XNJ on preventing autophagy

**Fig. 5:**
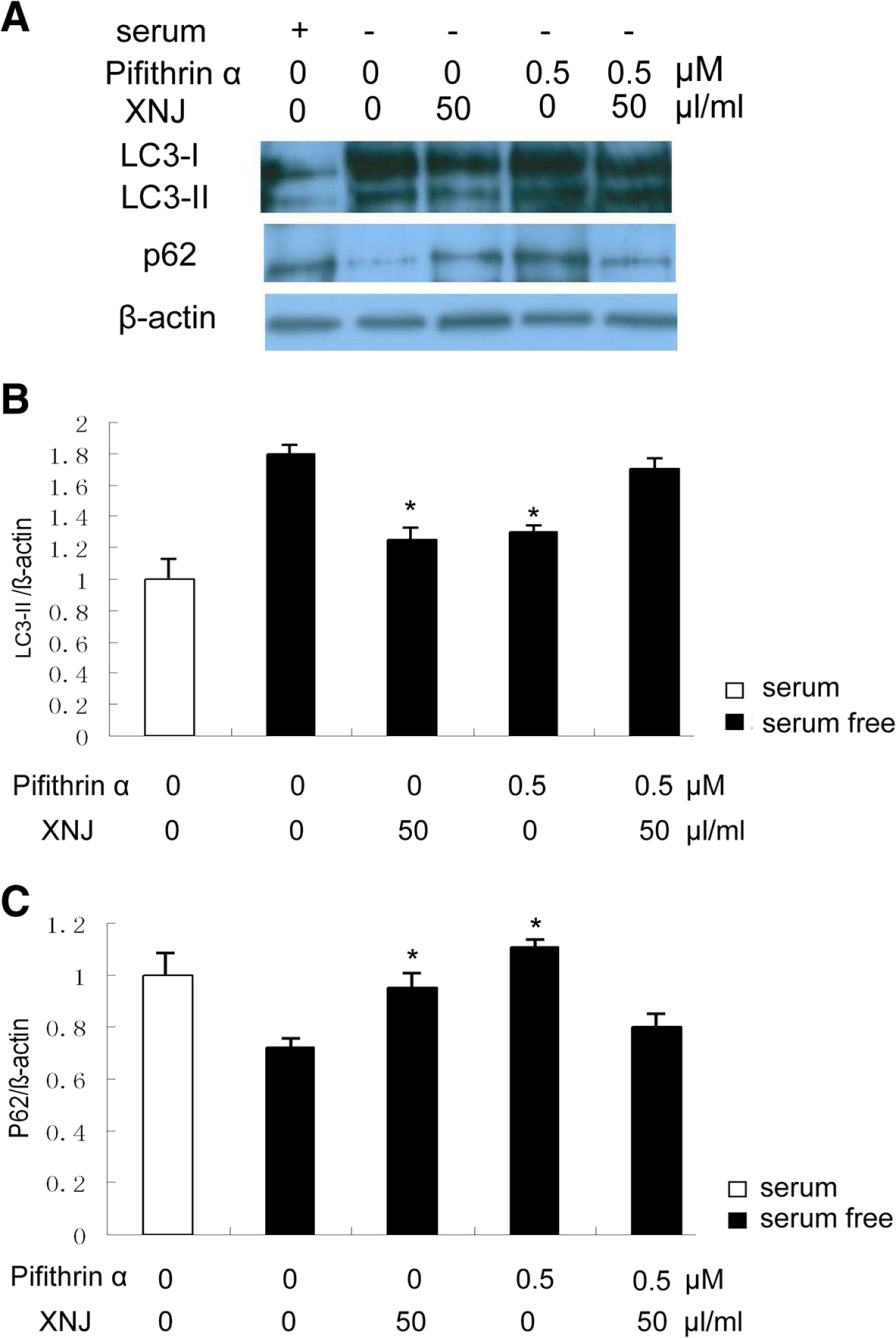
p53 is responsible for the effects of XNJ on preventing autophagy. **a** Suppression of p53 by p53 inhibitor significantly reduced the effects of XNJ on the autophagy. PC12 cells were cultured in serum-free condition and stimulated with p53 inhibitor in the absence or presence of XNJ, the expression levels of LC3 and p62 were analyzed using Western blot assay. **b** Comparison of the density of LC3-II/β-actin band among the experimental groups was showed. **c** Comparison of the density of p62/β-actin band among the experimental groups was exhibited. *, *p* < 0.05 compared with the serum-free condition group

To determine whether p53 played a role in the effects of XNJ on autophagy, PC12 cells were cultured in serum-free condition and stimulated with p53 inhibitor in the absence or presence of XNJ, the expression levels of LC3 and p62 were analyzed using Western blot assay. p53 inhibitor efficiently reduced LC3-II and increased p62 protein expression of the PC12 cells in serum-free condition (Fig. 5a). Interestingly, suppression of p53 by p53 inhibitor significantly reduced the effects of XNJ on the LC3-II and p62 expression of PC12 cells in serum-free condition (Fig. 5b). This suggests that p53 is responsible for the effects of XNJ on preventing autophagy of PC12 cells in serum-free condition.

### XNJ attenuates autophagy induced by cerebral ischemia

**Fig. 6:**
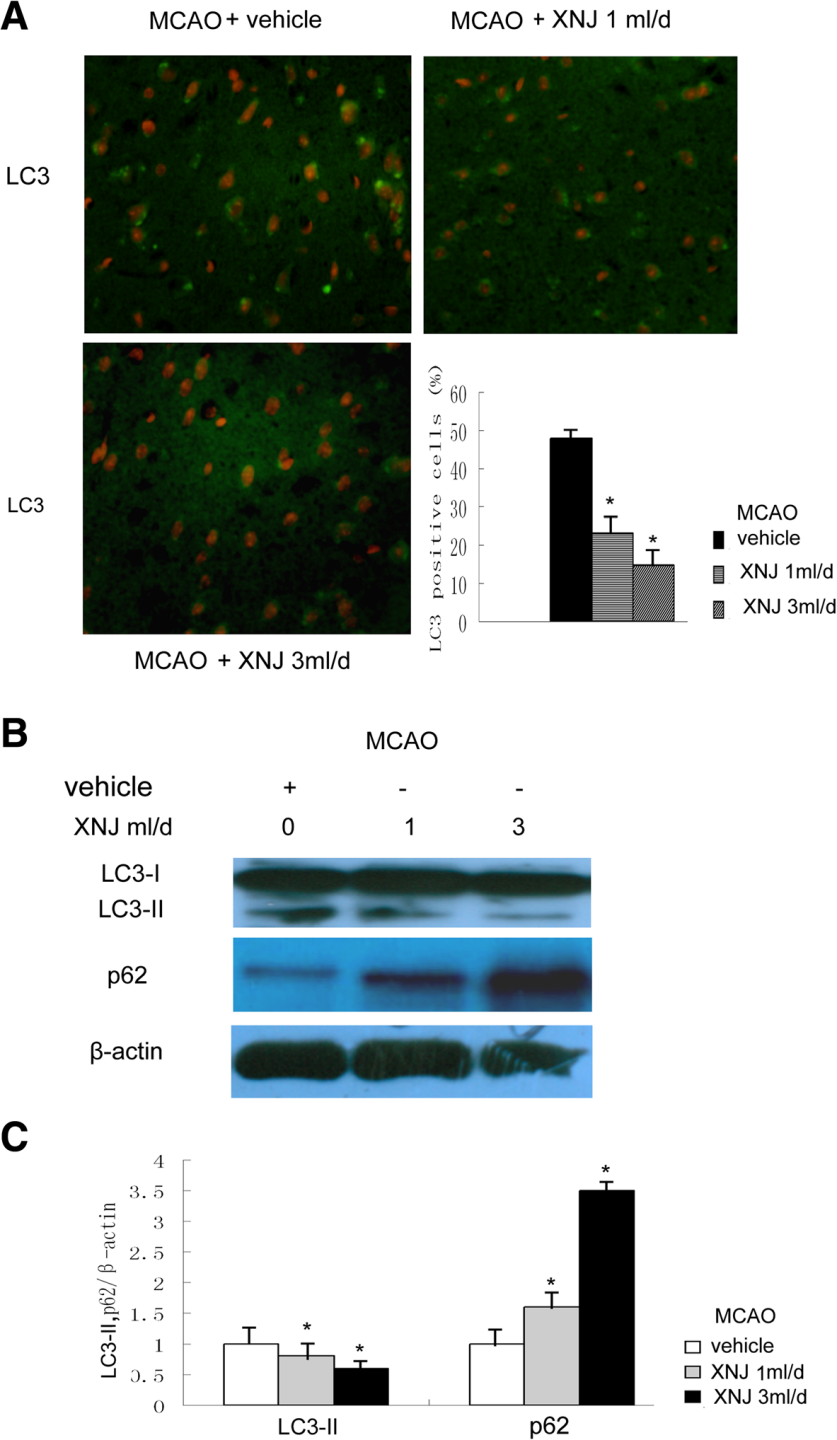
XNJ attenuated autophagy induced by cerebral ischemia. **a** The LC3-positive cells were analyzed using immunofluorescence 1 d after MCAO (upper panel, original magnification, 200×), and comparison of the percentage of LC3 positive cells among the experimental groups was exhibited (lower panel). Data are mean ± SEM of values obtained from 6 brains. **b** The levels of LC3 and p62 were analyzed by Western blot analysis. **c** Comparison of the density of LC3-II, p62/β-actin band among the experimental groups was showed. Data are the mean ± SEM of values obtained from 6 brains. *, *p* < 0.05, compared with the group of MCAO rats treated with vehicle

To explore the effects of XNJ on autophagy induced by cerebral ischemia, LC3 staining was used to identify autophagy cells in the parietal cortex 1 d after MCAO. There was an increase in LC3-positive cells in the group of MCAO rats treated with vehicle, whereas brain sections from the MCAO rats treated with XNJ showed very few LC3-positive cells. Quantitative analysis of LC3-positive cells (Fig. 6a) revealed that LC3-positive cells in the ischemic cortex reduced from 52 ± 3.1 % in the group of MCAO rats treated with vehicle to 24 ± 2.7 %, 13 ± 2.9 % in the group of MCAO rats treated with XNJ. These results suggest that the increase in LC3-positive cells in the ischemic cortex was inhibited by XNJ. In order to further characterize the anti-autophagy effects of XNJ, we examined the changes in LC3 and p62. The Western blot analysis was performed 1 d after ischemia to measure LC3 and p62 levels. The group of MCAO rats treated with vehicle showed a robust increase of LC3-II and reduction of p62 in the ischemic cortex. In contrast, LC3-II was attenuated and p62 was increased in the group of MCAO rats treated with XNJ (Fig. 6b). The quantitative analysis revealed that XNJ significantly decreased LC3-II and increased p62 levels after MCAO compared with the group of MCAO rats treated with vehicle (Fig. 6c). These results suggest that XNJ inhibited autophagy induced by cerebral ischemia.

### XNJ inhibited p53 and DRAM expression induced by cerebral ischemia

**Fig. 7:**
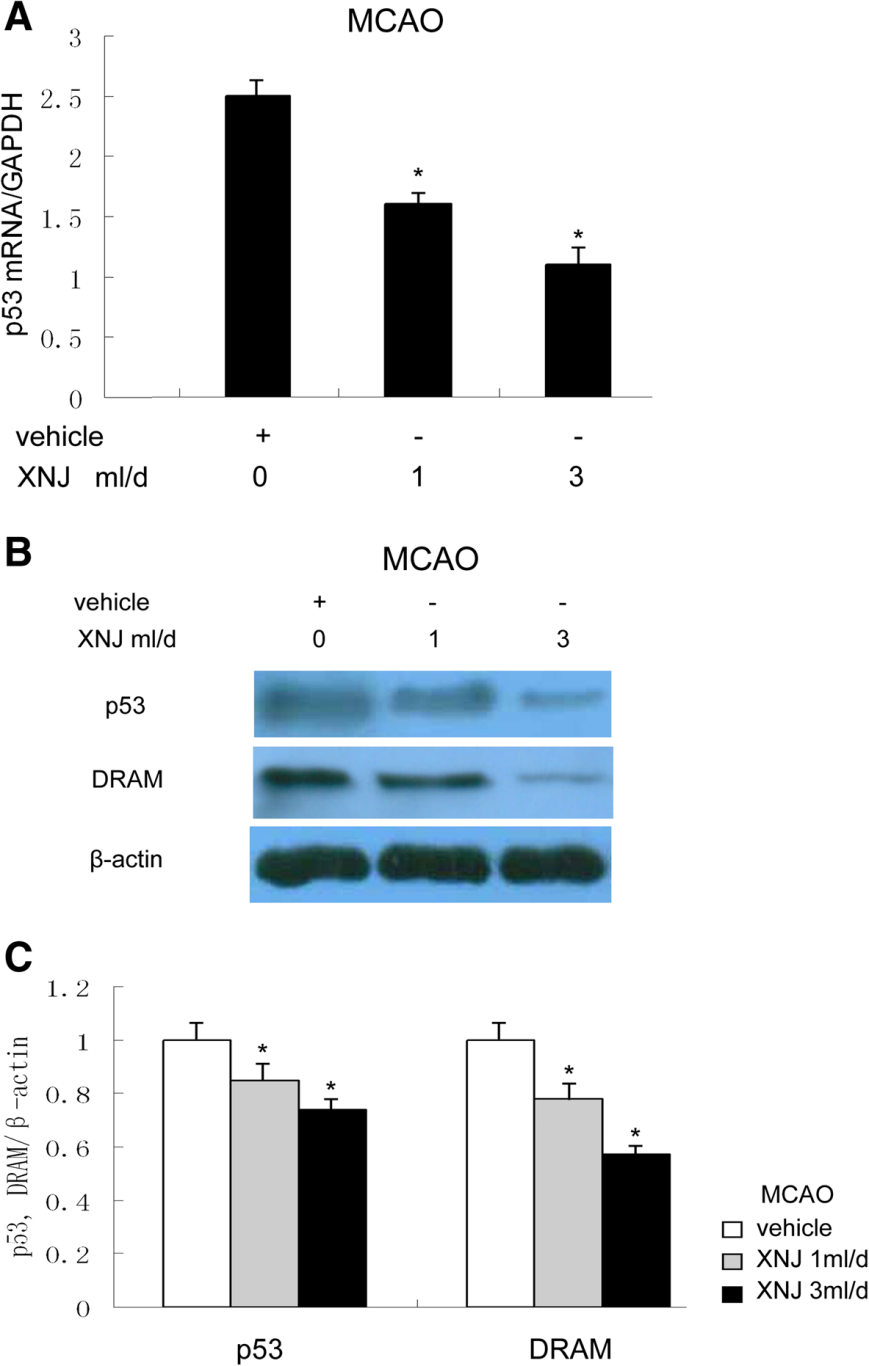
XNJ inhibited p53 and DRAM expression induced by cerebral ischemia. **a** Levels of p53 mRNA in the ipsilateral cortex were analyzed by real-time RT-PCR. **b** Levels of p53 and DRAM in the ipsilateral cortex were analyzed by Western blot. **c** The density of p53 and DRAM/β-actin band among the experimental groups was compared. Data are the mean ± SEM of values obtained from 6 brains. *, *p* < 0.05, compared with MCAO rats treated with vehicle

To determine whether the anti-autophagy activity of XNJ correlates with the expression of p53 and DRAM in a rat MCAO model, we examined the effect of XNJ on expression of p53 and its downstream effector DRAM in the ischemic cortex 1 d after MCAO. As shown in Fig. 7a, Real-time RT-PCR assay showed that XNJ significantly decreased the mRNA expression of p53 in the ischemic cortex after MCAO compared with the group of MCAO rats treated with vehicle. Western blot analysis demonstrated that XNJ significantly decreased the expression of p53 and DRAM in the ischemic cortex after MCAO compared with the group of MCAO rats treated with vehicle (Fig. 7b). The quantitative analysis revealed that XNJ significantly decreased p53 and DRAM levels compared with the vehicle group (Fig. 7c). These results suggest that XNJ inhibited p53 and DRAM induced by cerebral ischemia.

## Discussion

Although previous studies suggest that XNJ is protective, the effects and mechanisms of XNJ on the autophagy have yet to be investigated. The aim of this study was to further investigate whether the p53-DRAM pathway is involved in the effects of XNJ on autophagy. The major findings of the present study were: (1) XNJ prevents autophagy in experimental stroke. (2) The inhibition of autophagy via p53-DRAM signaling pathway is an important mechanism of protection by XNJ. These findings suggest that the dysfunction of the p53 could be a causative event leading to the autophagy, and that p53-DRAM signaling might be a key target for drug development and anti-autophagy therapy. The intervention of p53-DRAM signaling by pharmacological agents may represent a targeted and mechanism-based therapeutic strategy against brain damage following stroke.

An important finding revealed by the current study is that XNJ has an anti-autophagy effect. Starvation is the most commonly studied condition that induces autophagy [17]. To study the mechanism of autophagy, we established an autophagy model induced by serum-free condition, in which the PC12 cells were exposed to serum-free media 2 days. In serum-free condition, the autophagy was detected by a significant change of autophagy markers, including LC3, which is closely correlated with the extent of autophagosome formation [18] and p62, which is selectively incorporated into autophagosomes through direct binding to LC3 and efficiently degraded by autophagy [19]. In addition, we have found that in this model, gene expression of the autophagy was up-regulated, consistent with the notion that the DRAM family proteins predetermine the susceptibility of the cell to a given autophagic and apoptotic stimulus [8]. These observations indicate that autophagy induced in serum-free condition and cerebral ischemia could serve as an effective model system for screening potential agents in research. These results are in keeping with previous studies indicating that the serum-deprived PC12 cells show both autophagic and apoptotic features [20]. Recent evidence suggests that excessive autophagy results in neuronal cell damage [21–23]. This study describes the anti-autophagy effects of XNJ, in serum-free condition and cerebral ischemia, characterized by the reduction of LC3 and the up-regulation of p62. The inhibition of autophagy by XNJ improves cell survival, which provides a novel explanation for the protective effects of XNJ that benefit the nervous system.

An important mechanism revealed by the current study is that p53-DRAM signaling pathway is involved in the effects of XNJ on autophagy. p53 is a key regulator of cellular response to various stresses [6], and it performs its function primarily as a transcription factor, controlling the expression of a number of target genes [24]. Recent studies have shown that p53 has a dual role in the regulation of autophagy [7, 25], acting as a positive regulator of autophagy via its transcriptional activity and as a negative regulator of autophagy via its cytoplasmic functions [26]. Thus, the present work for the hypothesis that XNJ may regulate the p53 transcriptional activity was obtained by examining luciferase activity of p53 promoter. In agreement with our results, as positive control, pifithrin α, which is a synthetic inhibitor of p53-induced transcriptional activiation [27], also inhibits p53 transcriptional activity. As a transcription factor, p53 transactivates autophagy inducers DRAM. Our data provided by RT-PCR and Western blot analysis showed that XNJ reduced the expression of p53 and its target autophagy gene DRAM in serum-free condition PC12 cells and cerebral ischemia. Consistent with down-regulation of p53 and DRAM, we observed the anti-autophagy effect of XNJ on PC12 cells in serum-free condition and cerebral ischemia. To further determine the role of p53 transcriptional activity in the anti-autophagy effect of XNJ, our experiments with suppression of p53 transcriptional activity by p53 inhibitor have provided more direct evidence showing that the alteration of p53 transcriptional activity induced by serum-free and cerebral ischemia causally links to the autophagy and anti-autophagy effects of XNJ depending on p53-DRAM signaling pathway.

The present study has several important clinical implications. First, since excessive activation of autophagy contributes to neuronal death in cerebral ischemia [5], it promotes attractiveness for anti-autophagy therapy. Current study suggests that the autophagy should be a new target in the treatment of cerebral ischemia [28]. Thus, XNJ with anti-autophagy activity can be applied for therapies in stroke. Second, some reports showed that p53-DRAM signaling pathway has been associated with cell death [29], and that p53 inhibitor administration may be effective in the treatment of an animal model of stroke [30]. Current report suggests that targeting the p53 pathway represents a potential novel neuroprotective strategy to combat ischemic brain [31]. Hence, regulation of p53 signaling by XNJ may provide a new approach to the treatment of brain diseases. Third, our previous report showed that XNJ contained some small molecules including muscone, borneol and camphor [32], unlike large molecule agents, such as therapeutic antibodies or neurotrophic factors lack of transport across blood brain barrier [33]. There comes an innate advantage for XNJ to become therapeutic agents for brain diseases. Our findings are therefore of considerable therapeutic significance and provide the novel and potential application of XNJ for the treatment of brain diseases.

Since XNJ has various active ingredients, such as muscone which has been shown to exert neuroprotection via FAS pathway [32], it exerts the anti-autophagy effect through a multi-component and multi-target way. Furthermore, XNJ could act through a number of different mechanisms since p53 is involved in a number of cellular processes leading to cell death. Therefore autophagy prevention via p53-DRAM pathway is an important but not the only mechanism of protection by XNJ. Further studies are required in extension of the present observations in order to investigate other significant mechanisms regarding XNJ in the inhibition of autophagy.

## Conclusions

Our study suggests: (1) XNJ has an anti-autophagy effect; (2) The inhibition of autophagy via p53-DRAM signaling pathway is an important mechanism of protection by XNJ; (3) The intervention of p53-DRAM signaling by key components of pharmacological agents in this pathway may represent a targeted and mechanism-based therapeutic strategy against brain damage following stroke.
